# Bilateral adrenal gland haemorrhage: an unusual cause

**DOI:** 10.1530/EDM-14-0058

**Published:** 2014-08-01

**Authors:** Durgesh Gowda, Vasant Shenoy, Usman Malabu, Donald Cameron, Kunwarjit Sangla

**Affiliations:** 1 Department of Diabetes and EndocrinologyThe Townsville HospitalTownsville, QueenslandAustralia; 2 Department of SurgeryThe Townsville HospitalTownsville, QueenslandAustralia

## Abstract

Our patient had drainage of a large amoebic liver abscess. This got complicated by a severe degree of hypotension, which required aggressive fluid resuscitation and hydrocortisone support. Computerised tomography (CT) of the abdomen revealed bilateral adrenal gland haemorrhage (BAH) resulting in primary adrenal gland failure, which was the cause for hypotension. Patient was on long-term warfarin for provoked deep vein thrombosis of lower limb, which was discontinued before the procedure. Thrombophilia profile indicated the presence of lupus anticoagulant factor with prolonged activated partial thromboplastin time (aPTT). Patient was discharged on lifelong warfarin. This case emphasises the need for strong clinical suspicion for diagnosing BAH, rare but life-threatening condition, and its association with amoebic liver abscess and anti-phospholipid antibody syndrome (APLS).

**Learning points:**

- Recognition of BAH as a rare complication of sepsis.
- APLS can rarely cause BAH.

## Background

Bilateral adrenal gland haemorrhage (BAH) is a rare complication of bacterial sepsis (Waterhouse–Friderichsen syndrome). We report a case of BAH that occurred 3 days after percutaneous drainage of amoebic liver abscess. Our patient was also on warfarin for unknown procoagulant disorder. His aPTT was prolonged. Further investigation revealed positive for lupus anticoagulant factor suggestive of anti-phospholipid antibody syndrome (APLS), which can rarely cause BAH (1). This case provides an opportunity to discuss the relationship between BAH and amoebic liver abscess, which has been described only once in English literature (2), and to investigate on BAH caused by APLS.

## Case report

A 40-year-old male was referred to our surgical unit for management of large liver abscess. He had constant right upper quadrant abdominal pain, fever, rigour, sweats, diarrhoea and 5 kg weight loss. He had a history of travelling frequently to Ecuador for the last 3 years. The last trip was a month ago. Computerised tomography (CT) of the abdomen revealed 8.2×5.6 cm size abscess in the liver with other viscera being normal including adrenal glands. He was on warfarin for the last 3 years for single provoked deep vein thrombosis (DVT) of lower limb from travelling. He admitted non-compliance in taking warfarin. No details on procoagulant disorder were available. Stool sample revealed presence of *Entamoeba hartmanni*, *Blastocystis hominis* and *Endolimax nana*. Entamoeba histolytica antibody titre was 512 (>1:256). He was commenced on ceftriaxone and paromomycin. Warfarin was withheld and s.c. heparin was administered as bridging. He successfully underwent percutaneous drainage of 250 ml pus under imaging guidance. The aspirate confirmed the presence of Entamoeba histolytica.

**Figure 1:**
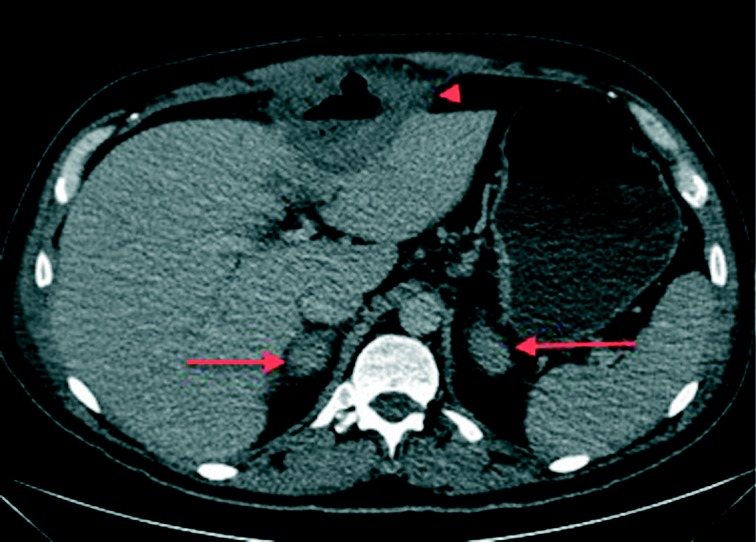
Axial view of CT adrenals revealing haemorrhagic adrenal glands (arrows) and drained abscess (arrow head).

**Figure 2:**
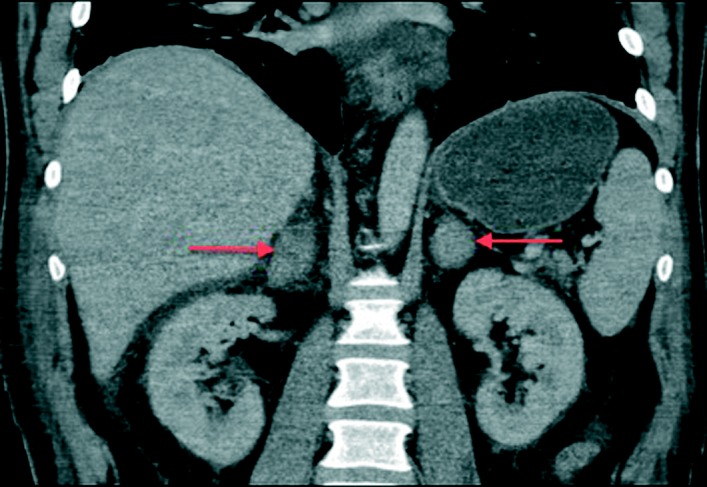
Coronal view of CT adrenals revealing haemorrhagic adrenal glands (arrows).

After initial recovery, he had clinical deterioration on day 3 after the procedure with significant hypotension (with systolic blood pressure ranging between 80 and 90 mmHg) and sinus tachycardia. He was afebrile and did not respond to initial i.v. fluid therapy and biochemistry showed low serum sodium of 127 mmol/l (135–145 mmol/l), normal renal function and improving cholestatic liver function tests. He required activation of medical emergency team and was given i.v. hydrocortisone after appropriate blood tests and repeated imaging. Urgent CT abdomen revealed significant reduction in the size of the abscess along with significant bilateral adrenal enlargement with haemorrhages (Figs 1 and 2). In keeping with this, the assays confirmed an extremely low cortisol level of 15 nmol/l (200–650 nmol/l) and undetectable aldosterone level (<70 pmol/l) suggestive of primary adrenal gland failure.

He recovered well from acute adrenal insufficiency. He was discharged on divided doses of hydrocortisone as 10 mg, 4 mg, 4 mg and fludrocortisone 100 μg once daily along with education on sick day management. However, he represented within a week with the swollen leg and was confirmed to have DVT and pulmonary emboli. Coagulation studies revealed prolonged aPTT that remained prolonged after mixing study. Further investigations revealed the presence of the lupus anticoagulant factor with low titres of anticardiolipin antibody and β2 glycoprotein suggestive of lupus anticoagulant disorder.

He was transferred to the haematology unit for further care and required warfarin, corticosteroid and mineralocorticoid lifelong on discharge.

## Discussion

The incidence of spontaneous adrenal gland haemorrhage from autopsy studies is ∼0.14–1.1%. To the best of our knowledge, BAH has only been reported once in English literature in a patient with amoebic liver abscess resulting in adrenal insufficiency (2). Herein, we report the second case.

Elevated ACTH level is the proposed hypothesis as pathophysiology in the context of sepsis from amoebic liver abscess (2). This is based on animal studies (3). In this study, normal rats that received 60 μg/day of ACTH for 16 weeks had a severe degree of adrenal gland haemorrhage and vacuolisation. Elevated ACTH level is postulated to increase the arterial blood flow to the adrenal glands, in excess of the venous drainage contributing to the disruption of the delicate architecture and haemorrhage (4). The usual time frame for clinical presentation with hypotension and hyponatraemia is 48–72 h as was in our case (5).

The common causes for spontaneous adrenal gland haemorrhage include severe sepsis, burns, trauma and major surgery. The other causes include adrenal tumours both primary and secondary, abdominal tumours and pregnancy, and spontaneous adrenal gland haemorrhage may occur as a post-operative complication (6).

Minor surgical procedures do not usually lead to spontaneous adrenal gland haemorrhage (5).

APLS has been noted to cause spontaneous adrenal gland haemorrhage (4). In our patient, lupus anticoagulant was positive with unremarkable levels of anticardiolipin antibody and β2 glycoprotein, and this was confirmed on repeated testing. However, in our patient, adrenal glands on the initial imaging for the liver abscess were well visualised with normal appearances and he had no related history.

The proposed hypothesis (7) for APLS causing BAH involves a high level of cholesterol in adrenal gland. This attracts the anti-phospholipid antibodies into the adrenal gland, which in turn leads to thrombosis and haemorrhage.

CT imaging without contrast can diagnose acute adrenal bleeding as low or mixed attenuation in the centre. The usual appearance is central low attenuation with peripheral enhancement representing preserved adrenal tissue (8). The bleed continues to expand giving the gland an oval or round configuration. MRI if performed reveals a high T1 signal intensity. Over time, adrenal haemorrhage appears as a pseudocyst with peripheral calcification and eventually results in atrophy of the gland, though this is another feature. Duration for this atrophy is not well described.

BAH requires empirical treatment initially with i.v. corticosteroids, usual daily doses being in the order of 150–200 mg hydrocortisone or equivalent alternatives (4). The doses are reduced over few days to replacement regimens of 15–30 mg hydrocortisone or equivalence in divided doses coupled with mineralocorticoid replacement. This needs to be coupled with education for sick day management and increase in steroid doses as well as medical alert tags to be carried on person for emergency use.

## Patient consent

Verbal consent was obtained and the patient is now deceased.
